# A Novel *In Vitro* Platform Development in the Lab for Modeling Blast Injury to Microglia

**DOI:** 10.3389/fbioe.2022.883545

**Published:** 2022-07-12

**Authors:** Dasen Xu, Nu Zhang, Sijie Wang, Yawei Yu, Pan Zhang, Yulong Li, Hui Yang

**Affiliations:** ^1^ School of Aeronautics, Northwestern Polytechnical University, Xi’an, China; ^2^ Center of Special Environmental Biomechanics and Biomedical Engineering, Northwestern Polytechnical University, Xi’an, China; ^3^ School of Life Sciences, Northwestern Polytechnical University, Xi’an, China

**Keywords:** dynamic compression stress, primary microglia, device development, *in vitro* cell model, blast injury

## Abstract

Traumatic brain injury (TBI), which is mainly caused by impact, often results in chronic neurological abnormalities. Since the pathological changes *in vivo* during primary biomechanical injury are quite complicated, the in-depth understanding of the pathophysiology and mechanism of TBI depends on the establishment of an effective experimental *in vitro* model. Usually, a bomb explosive blast was employed to establish the *in vitro* model, while the process is complex and unsuitable in the lab. Based on water-hammer, we have developed a device system to provide a single dynamic compression stress on living cells. A series of amplitude (∼5.3, ∼9.8, ∼13.5 MPa) were generated to explore the effects of dynamic compression loading on primary microglia within 48 h. Apoptosis experiments indicated that primary microglia had strong tolerance to blast waves. In addition, the generation of intercellular reactive oxygen species and secretory nitric oxide was getting strongly enhanced and recovered within 48 h. In addition, there is a notable release of pro-inflammatory cytokine by microglia. Our work provides a reproducible and peaceable method of loading single dynamic compression forces to cells *in vitro*. Microglia showed an acute inflammatory response to dynamic loadings, while no significant cell death was observed. This insight delivers a new technological approach that could open new areas to a better understanding of the mechanism of cell blast injuries.

## Introduction

Traumatic brain injury (TBI) is an international health concern, which results in chronic neurological abnormalities including cognitive deficits, emotional disturbances, and motor impairments (Maas et al., 2000; Marshall, 2000; Doppenberg et al., 2004; Thompson et al., 2005). The effective treatment is limited due to the complex pathological processes of primary and secondary injury to the central nervous system (CNS) that follow TBI (Schmidt et al., 2005; Atif et al., 2009). Primary injury includes damages induced by mechanical impact on the brain (Graham et al., 2000; Marshall, 2000). Secondary injury results from processes triggered by primary insults, such as oxidative/nitrative stress, acute inflammation, or apoptosis by microglial response (Farkas et al., 1998; Marshall, 2000; Bao et al., 2010).

The *in vivo* TBI experimental model had been successfully built depending on the fluid-percussion model which was designed by Denny and Russell (Cernak et al., 2011; Rubovitch et al., 2011; Huber et al., 2013). The model generates a controllable impact on an animal’s head (Denny-Brown and Russell, 1941). On the other hand, various *in vitro* impact injury experimental models have also been developed for understanding the mechanical stimuli during an impact loading as well as subsequent biological responses of cells (Morrison et al., 2011). When exploring the cell behaviors after dynamic stress is applied, including the biological responses and establishing cell stress–strain relationships, the challenge is how to properly apply a controllable force on the monolayer or single-cell [capture its real-time strain field at a single cell scale (i.e., $10^{-5}m$ ) for mechanical behaviors] (Patterson, 2020). This challenge is mainly related to the dynamic condition, which is totally different from static or quasi-static conditions. With atomic force microscopy (AFM), magnetic twisting cytometry (MTC) (Trepat et al., 2004), and uniaxial stretching rheometer (USR) (Desprat et al., 2005), it is not difficult to simulate a static or quasi-static condition to explore the mechanic of cell behaviors after applying forces. While for the dynamical loading process, the most useful device to generate controllable and purely dynamical stress is the split Hopkinson’s pressure bar (SHPB). While for a monolayer or single cell, it is so hard to find two such small or soft bars (the length scale for a single cell is about $10^{-5}m$ , and the young’s modulus of a single cell is usually below $10^{0}MPa$ ), which means it cannot reach a stress equivalent condition in this process. Also, it is hard to hold such a single cell between 2 bars. Therefore, various reasonable assumptions were proposed, mostly based on the research-scale perspective and the corresponding mechanical methods, including the mechanical and the biological methods (Moeendarbary and Harris, 2014; Hao et al., 2020). Despite various experimental models that have been built for tissues or monolayers, there are still challenges in applying dynamic pressure loading on a single cell *in vitro* (Shepard et al., 1991; Cernak et al., 2011; Rubovitch et al., 2011; Huber et al., 2013). Interference of various types of stress loading (compress, shear, and tensile, etc.) simultaneously makes the data recording and analysis complicated. In addition, an open-field underwater blast or shock tube, which is a common method to keep cells exposed to the shock wave, needs special experimental conditions (Sawyer et al., 2017a; Sawyer et al., 2018). Therefore, generating acute dynamic loadings with physiologically relevant length-scales and time-scales under normal cell culture conditions is a major limitation in understanding the biomechanical mechanism of how cells respond to stress loading (Shepard et al., 1991; Moeendarbary and Harris, 2014).

Acute inflammatory responses of TBI include alterations in the permeability of the blood–brain barrier, infiltrations, and accumulations of polymorphonuclear leukocytes and hematogenous macrophages (Farkas et al., 1998; Morganti-Kossmann et al., 2001; Raghupathi, 2010). Subsequently, the produced pro-inflammatory cytokines and reactive free radicals by injured microglia change the vascular permeability to cause brain edema formation and exacerbate the pathologic injury process (Scholz et al., 2007). In addition, acute inflammatory events can also lead to the production of harmful reactive oxygen species, nitric oxide, and other detrimental molecules causing nervous cell necrosis and apoptosis (Shohami et al., 1994; Nguyen et al., 2010). As brain-resident macrophages, microglia, are activated rapidly during TBI, they are involved in neuroinflammation, secondary brain injury, and CNS repairment (Hangzhe et al., 2017). In addition, studies have shown that microglia are sensitive to primary overpressure waves and secrete a variety of reactive species and cytokines (Kane et al., 2012; Clark et al., 2019). However, it remains unclear whether these changes were induced directly by the dynamic pressure loading or caused indirectly by the damage of brain tissue (Readnower et al., 2010).

Here, based on the theory of dynamic loading in fluid-filled tubes, we designed and developed a dynamic pressure loading system for cells *in vitro*. The system could provide a controllable, single-pulse dynamic compression loading into the cell medium, with the collecting of the loading parameters directly. Furthermore, the potential functional acute response of primary microglia after dynamic pressure loading was investigated by analyzing the cell viability and oxidative/nitrative stress as well as inflammatory response.

## Materials and Methods

### Basic Principle and Design of the Dynamic Pressure Loading System

**FIGURE 1:**
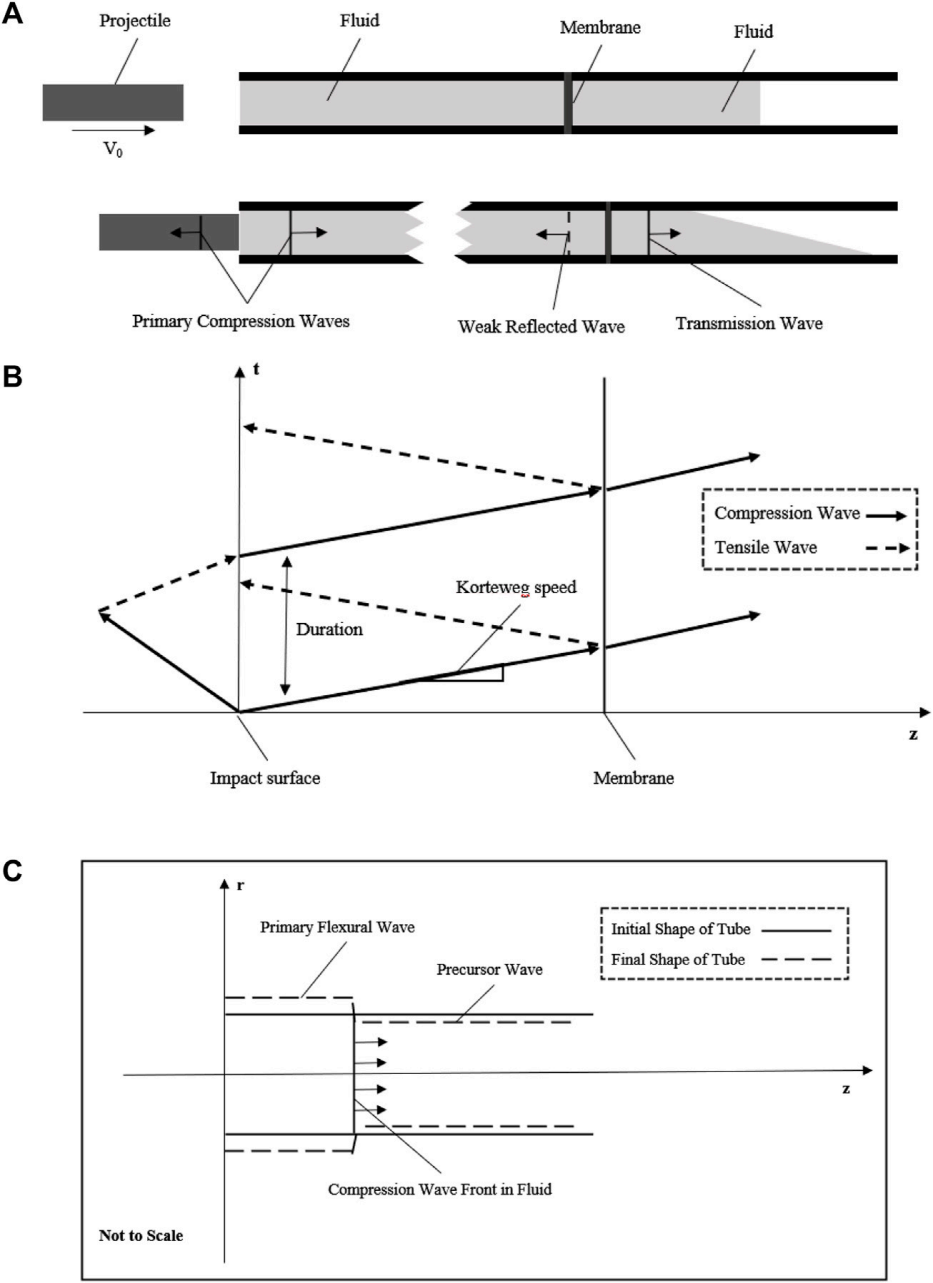
Schematic diagram showing the basic principle of the fluid–structure interaction model. **(A)** Projectile with initial velocity impacts the fluid to generate a compression wave in the fluid; the compression wave across the membrane at the middle of the tube with weak reflected wave and transmission wave into the extended fluid; **(B)** generation and propagation of compression wave with the space–time plot in this simplified model; **(C)** fluid–structure interaction of this tube model to show the axial motion due to the Poisson’s effect.

Dynamic pressure loading inside a fluid-filled tube, modeled as a “water-hammer,” is a well-known problem in power and process plants (Wylie et al., 1993). Based on this theory, we could generate a weak shock wave [the “weak” signifies that the thermal energy generated by impact compression is small compared to the total internal energy of the fluid (Smith, 1973)], which would propagate in fluid matter at an acoustic speed, once a projectile with an initial velocity [ $\left(V_{0}\right)$ ] impacts a fluid column in the tube. A schematic diagram of this impact process is presented in Figure 1A. A fundamental assumption is that there is no cavitation of fluid column separation occurs, and the cross-section change is within the elastic range. Then, to describe the pressure change of this weak shock wave, a Joukowsky equation is a perfect approximation for predicting the maximum pressure due to this water-hammer impact (Joukowsky, 1900; Walters and Leishear, 2018):

$$\begin{array}{c} P\left(0\right)=\frac{{\left(\rho c\right)}_{f}{\left(\rho c\right)}_{p}}{{\left(\rho c\right)}_{f}+{\left(\rho c\right)}_{p}}V_{o} \end{array},$$ (1)

Where ${\left(\rho c\right)}_{f}$ is the fluid acoustic impedance and ${\left(\rho c\right)}_{p}$ is the projectile acoustic impedance. In most cases, the impedance of projectile is much higher than that of fluid, so Eq. 1 could be rewritten as:

$$\begin{array}{c} P\left(0\right)\approx {\left(\rho c\right)}_{f}V_{o} \end{array}.$$ (2)

Deshpande et al. (2006) had theoretically and experimentally confirmed that the pressure would rise to its maximum value almost instantaneously and then decreases at a nearly exponential rate. Then, the equation to approximately describe the pressure change of this weak shock wave could be written as:

$$\begin{array}{c} P=P\left(0\right)\exp\left(-t/{\left(\frac{m}{\rho c}\right)}_{p}\right) \end{array},$$ (3)

Where the subscript p represents projectile. Here, we know that we could simulate an underwater shock wave or generate a dynamic compression stress wave in fluid based on such a water-hammer model. Due to the aim is simulating a single compression stress wave environment, we designed a membrane setting that has a very closer acoustic impedance to fluid to reduce the reflected stress wave as possible as shown in Figure 1A.

While in this model we have not taken the tube effects into consideration. Then, we need to know to evaluate the fluid–structure interaction problem. The acoustic speed $\left(c_{o}\right)$ of this weak shock wave could be calculated by the sound speed equation:

$$\begin{array}{c} c_{0}={\left(\frac{K_{f}}{\rho_{f}}\right)}^{\frac{1}{2}} \end{array},$$ (4)

Where $K_{f}$ is the fluid bulk modulus and $\rho_{f}$ is the density of the fluid. Here, the fluid is considered compressible to allow the shock wave propagation. However, classical water-hammer theory did not neglect the coupling motion between the fluid and tube during this wave propagation process (Skalak, 1956; Tijsseling, 1996), which means that despite the fluid being considered compressible, the deformation of the tube wall should be taken into account. Here, a general solution of wave celerity has been derived by Hutarew (1973) to describe this interaction:

$$\begin{array}{c} c_{f}=1/\left(\rho_{f}\left(\frac{1}{K_{f}}+\frac{1}{A}\frac{\delta A}{\delta P}\right)\right) \end{array},$$ (5)

Where A is the cross-section area of the channel, P is the total pressure, $c_{f}$ is fluid wave propagation celerity, and δ is a small change sign. Although a series of complex theoretical processing was performed during this fluid–structure interaction process, Korteweg had proposed a quasi-one-dimensional model to predict the key physical effects, which then had been confirmed experimentally by Joukowsky and Tijsseling (Joukowsky, 1900; Tijsseling, 1996; Korteweg, 2010). Those research studies support that the key effects are the pressure generated by the acoustic wave in the water which is balanced by static stress in the surrounding tube, considering the purely elastic radial deflection uncoupled from the longitudinal motion (Junger and it, 1972; Howe, 1998). Then, a classical formula for wave celerity in an elastic tube had been first derived by Korteweg:

$$\begin{array}{c} c_{f}^{-2}=c_{o}^{-2}+\frac{\rho_{f}2R}{E_{s}h} \end{array},$$ (6)

Where R is the mean radius of the tube, $E_{s}$ is the elastic modulus of the tube, and h is the thickness of the tube. A more clearly writing to describe the coupling between the tube and fluid is as follows:

$$\begin{array}{c} c_{f}=c_{o}{\left(1+\left(\frac{c_{o}^{2}}{c_{s}^{2}}\right)\left(\frac{\rho_{f}}{\rho_{s}}\right)\left(\frac{2R}{h}\right)\right)}^{-0.5} \end{array},$$ (7)

Where $c_{s}$ is the acoustic speed of the tube and $\rho_{s}$ is the density of the tube. Moreover, a single non-dimensional parameter *β* is introduced (Shepherd and Inaba, 2009; Perotti et al., 2013) to determine the extent of fluid–structure coupling in this model:

$$\begin{array}{c} \beta =\left(\frac{c_{o}^{2}}{c_{s}^{2}}\right)\left(\frac{\rho_{f}}{\rho_{s}}\right)\left(\frac{2R}{h}\right) \end{array}.$$ (8)

Eq. 6 could be rewritten as the Moens–Korteweg wave speed equation as follows:

cf=c01+β. (9)

After that, a whole stress wave propagation process in fluid including the impact and membrane setting had been described in Figure 1B. This theoretical wave celerity could be used to compare with the experimental wave celerity to check whether this system works.

As the tube wall has a purely elastic radial deflection due to the stress wave, it may be workable to acquire the stress wave data with a non-contacted measuring method of the tube wall to reduce potential error. As R. Skalak had solved the initial problem of water-hammer, there are two waves, a primary flexural wave (hoop motion) accompanied by the shock wave in fluid and a precursor wave (weak axial motion) due to the Poisson’s effect as shown in Figure 1C. These waves had been observed by Shepherd and Inaba, (2009), and the experimental data had an agreement with the theory of R. Skalak. The primary flexural wave (hoop strain) propagates at the same velocity as the shock wave in fluid, while the precursor wave (weak axial strain) propagates at a much higher speed, the acoustic speed of the tube wall. Importantly, as the pressure change is exceedingly small due to this precursor, there is approximately only one wave propagating in the fluid. Since the initial and exact solutions of the water-hammer had already been given, once the dynamic pressure loading system meets the basic principle mentioned previously, the pressure in the fluid could be evaluated by the strain of the tube wall (Leishear, 2005; Leishear, 2006; Leishear, 2007).

After we have ensured the stress generation part, evaluation, and measuring methods, we need to consider how the cells will suffer from this compression stress. The length scale of the living cell is about $10^{1}\mu m$ , while this is too far smaller than the length scale of a tube or fluid column once designed at $10^{1}mm$ . So, it is reasonable to treat the isolated living cell as a particle which means cells would not have any effect on stress waves. Also, a uniform treatment would make sure the cells would suffer from the nearly same stress.

### The Construction of the Dynamic Pressure Loading System

According to the model we built, a dynamic pressure loading system was developed to generate a single compression wave on cells *in vitro* (Deshpande et al., 2006). The structure of the system is shown inFigure 2, which consists of gas gun, O-ring, cell tube, strain gauges, pressure transducer, and a hollow extended tube set. The gas gun was made by Northwestern Polytechnical University. The O-ring and cell tube were made of steel. The cell tube had a length, thickness, and diameter of 140 mm × 0.25 mm × 12 mm. The hoop strain of the cell tube during impact was measured in real-time by semiconductor strain gauges stuck on the surface. The pressure transducer (BoSiDe), with a working range of 0–15 MPa, was connected at the bottom of the cell tube to measure the pressure of the fluid inside.

**FIGURE 2:**
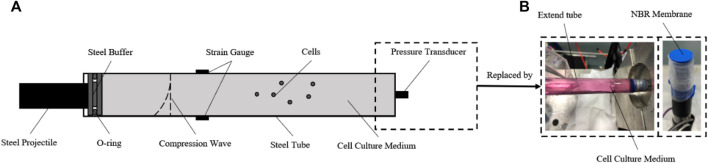
Construction of the dynamic pressure loading system. **(A)** Schematic diagram of the dynamic pressure loading system. For evaluation and calibration, the semiconductor strain gauges were stuck in the middle surface of the cell tube, and the pressure transducer was connected to the bottom of the cell tube; **(B)** for loading pressure to cells *in vitro*, the pressure transducer was replaced by the NBR membrane and an extra column of fluid in a hollow tube extended.

According to the aforementioned theories, a compression wave could be generated by an accelerated steel projectile impacting the O-ring. The amplitude, duration, and waveform of the compression wave could be adjusted by changing the parameters of the projectile (velocity, mass/length, and material) which is controlled reproducibly and precisely. After impact loading, the compression wave propagates in the cell medium, accompanied by a hoop tensile wave in the tube wall.

Importantly, to load a single impact wave within the fluid, an extra column of fluid in a hollow tube is connected after the cell medium, which helps the compression wave continue to propagate and reduces any reflex waves (Figure 2B). A nitrile butadiene rubber (NBR) membrane, which has a similar wave impedance to the cell medium, was used to separate the sterile cell medium and the extra fluid. For the cell experiment, the pressure transducer was replaced by this hollow tube after the pressure data acquisition was finished. The pressure within the medium in the tube could be calculated by the hoop strain of the cell tube (Skalak, 1956).

### Animals

Newborn C57BL/6J mice were obtained from the Medicine Laboratory Animal Center, Xi’an Jiaotong University, and housed in an environmentally controlled facility with food and water *ad libitum*.

### Primary Microglia Cultures

Primary microglia were obtained and isolated from C57BL/6J mice (Yu et al., 2019; Scheiblich et al., 2021). Brain cells were dissociated by mechanical shearing and trypsin and cultured with DMEM/F12 complete medium with PLL-coated culture flasks. After 10 days, the flasks containing brain cells and culture medium were shaken at 220 rpm for 40 min to collect primary microglia suspension, which would be re-cultured in six-well plates for subsequent treatment (Long et al., 2020).

### Microglial Cell Treatment

Primary microglia cells were suspended by 0.5% trypsin before each mechanical stimuli treatment. A 120-mm-long tube is fulfilled with the cell medium containing 3 × 106 microglia, and a 25-mm-long steel projectile was used to generate various levels of pressure. After treatment, microglia were transferred onto a 96-well plate immediately and cultured at different time points for subsequent analysis. The experimental time points 12, 24, and 48 h reflected the cumulative time. Microglia loaded in the tube with no pressure applied were used as control groups.

### Cell Survival Rate

The cell survival rate was assessed by cell counting kit-8 (CCK-8, Dojindo) following the manufacturer’s instructions. The optical density (OD) at 450 nm was detected using a microplate reader (Bio-Tek). The calculation method is as follows:

$$\begin{array}{c} Cell\;survival\;rate\left(\%\;Control\right)=\frac{OD\;value_{experiment}-OD\;value_{backgroud}}{OD\;value_{Control}-OD\;value_{backgroud}}\times 100\% \end{array}.$$ (10)

Also, a standard curve between the absorbance values at 450 nm and the number of cells in a 96-well plate had been tested according to the manufacturer’s instructions. A linear fitting of this standard curve was used to calculate the number of cells in each well of every group, to possibly reduce the influence of the number of cells changing.

### Caspase-3 Activity Assay

The caspase-3 activity was determined using Caspase 3 Activity Assay Kit following the manufacturer’s instructions (Beyotime). The results were recorded using a microplate reader at a wavelength of 405 nm. The detection samples were acquired by cell lysis and centrifugation at 4°C. This assay was based on the principle that Ac-DEVD-ρNA (acetyl-Asp-Glu-Val-Asp p-nitroanilide) is catalyzed by caspase-3 and then produces ρNA (p-nitroaniline). The absorbance of each sample at 405 nm was measured, and the caspase-3 activity was calculated in combination with the standard curve and protein concentration. The results are presented as the mean units/mg ± standard error of the mean (S.E.M) for every 10 k cells.

### Lactate Dehydrogenase Assay

Cell death was evaluated by quantifying the lactate dehydrogenase (LDH) using LDH Cytotoxicity Assay Kit (Beyotime) according to the manufacturer’s instructions. At each experimental time point, the cell culture was collected to test the accumulated released LDH. The results were presented in a form of accumulated LDH released for every 10 k cells with an absorbance at 490 nm. The calculation method is as follows:

$$\begin{array}{c} LDH=\left(\frac{OD_{experiment}-OD_{blank}}{number\;of\;cells_{experiements}}\right)/\left(\frac{OD_{Control}-OD_{blank}}{number\;of\;cells_{experiements}}\right) \end{array}.$$ (11)

### Detection of Intracellular Reactive Oxygen Species and Nitric Oxide Production

The intracellular ROS levels were measured using Reactive Oxygen Species Assay Kit (Beyotime) following the manufacturer’s instructions. Fluorescence was observed by fluorescence microscopy (Olympus) and recorded using a fluorescence spectrophotometer (Bio-Tek) (excitation wavelength of 485 nm and emission wavelength of 525 nm). The fluorescence intensity was normalized against the control wells and the number of cells:

$$\begin{array}{c} \mathrm{Relative}\;\mathrm{level}\;\mathrm{of}\;\mathrm{ROS=}\left(\frac{\mathrm{valu}e_{\mathrm{experiment}}\mathrm{-valu}e_{\mathrm{blank}}}{\mathrm{number of cell}s_{\mathrm{experiements}}}\right)/\left(\frac{\mathrm{valu}e_{\mathrm{control}}\mathrm{-valu}e_{\mathrm{blank}}}{\mathrm{number of cell}s_{\mathrm{control}}}\right) \end{array}.$$ (12)

Total NO production in the culture medium was determined by measuring the concentration of nitrate and nitrite, a stable metabolite of NO, using a modified Griess reaction by Total Nitric Oxide Assay Kit (Beyotime). OD at 540 nm was measured using a microplate reader, and the NO concentrations were calculated by comparing absorptions with the standard curve. The results were presented as a modified form (mean μmol/L ± S.E.M for every 10 k cells).

### Enzyme-Linked Immunosorbent Assay

ELISA kit (Neobioscience) was used to quantify the concentration of tumor necrosis factor-α (TNF-α) in the culture medium following the manufacturer’s instructions. OD at 450 nm was measured using an ELISA reader (Bio-Tek). The results were presented as a modified form (mean pg/mL ± S.E.M for every 10 k cells).

### Statistical Analysis

All biological data were expressed in the form of mean±S.E.M from at least three independent experiments ( n≥3 ). Biological results were analyzed by one-way ANOVA with Tukey’s *post hoc* test (the corresponding codes were programmed by R (v4.1.3) in IntelliJ IDEA, including analysis and plotting process). The comparisons of results between the experimental groups versus control groups (no comparison between the different experimental groups) were only within each experimental time point. A p -value of <0.05 was deemed to be statistically significant and is indicated in the figures by an asterisk. The P -values of <0.01 and <0.001 were indicated by two and three asterisks, respectively.

## Results

### Evaluation and Calibration of the Dynamic Pressure Loading System

**FIGURE 3:**
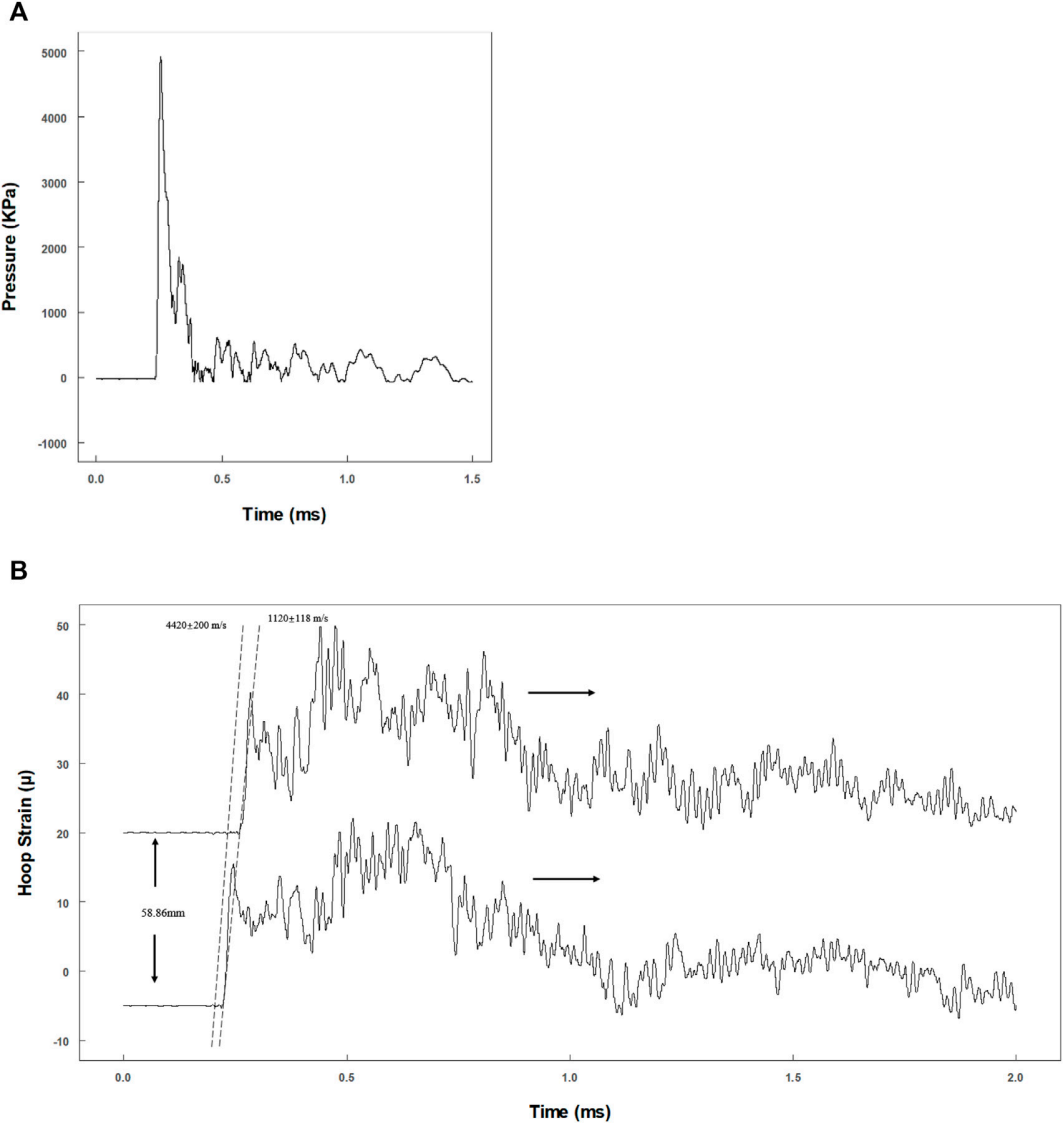
Evaluation of the dynamic pressure loading system. **(A)** Tracing data of pressure transducer at the end of the tube; **(B)** hoop strain tracings by two pairs of semiconductor strain gauges with 58.86 mm separation.

To evaluate whether the dynamic pressure loading system matches the theoretical model, the actual primary flexural wave speed needs to be calculated. A representative pressure data recorded by the pressure transducer are shown in Figure 3A. The pressure reached a peak within 20 μs and declined exponentially. The actual maximum pressure inside the fluid should be half of the max recording value, as the transducer was at the tube end (Veilleux and Shepherd, 2017).

Two pairs of strain gauges (distance: 58.9 mm) were used to record the time phase difference of the primary flexural wave in the tube wall (Figure 3B). Theoretically, the propagation velocity of the primary flexural wave (Korteweg wave celerity) should be much slower than that of the precursor wave (acoustic celerity in the tube) (Shepherd and Inaba, 2009). The velocities were 4,620 ± 200 m/s (precursor wave) and 1,120 ± 118 m/s (primary flexural wave) (Figure 3B), which are consistent with the theoretical velocities of 4,996 m/s (acoustic velocity in steel) and 1,201 m/s (Korteweg wave celerity) (Shepherd and Inaba, 2009).

**FIGURE 4:**
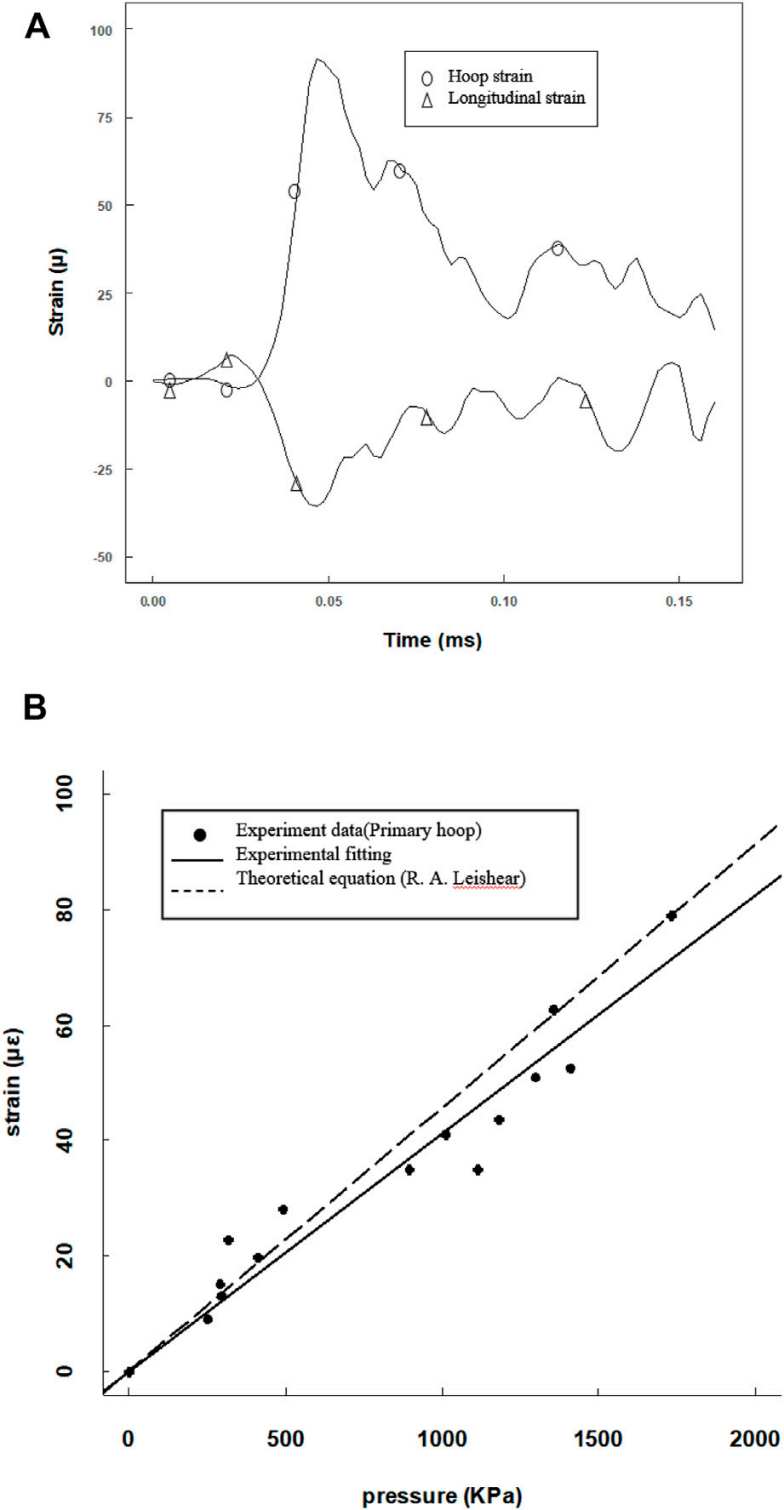
Calibration of the pressure amplitude from the pressure and strain data provided by the pressure transducer and hoop strain gauges. **(A)** Hoop and longitude strain tracings at the same position; **(B)** max pressure amplitude versus max hoop point plot with a linear fitting, compared with a theoretical curve.

Since the tube length was 120 mm, a 25-mm-long steel projectile was used to avoid waveform superposition. The projectile was accelerated by a gas gun to impact the cell medium, and an overview of the results is presented in Figure 4. Both the primary flexural and the precursor waves on the tube were recorded by the hoop and longitudinal strain gauges (Figure 4A). The motions in hoop and longitudinal were synchronous, and the ratio was nearly 0.3 consistent with the Poisson’s ratio. As the hoop strain was less than 2 mε, the hoop motion of the tube wall was elastic deformation. Here, the maximum hoop strain and maximum fluid pressure yielded a linear equation in calibrating the pressure amplitude (Figure 4B). The experimental data linearly fitted a constant of 0.0425 (R2 = 0.9805) which is consistent with the theoretical parameter of 0.0456 (Leishear, 2006; Leishear, 2007).

### Microglial Cell Viability Analysis

**FIGURE 5:**
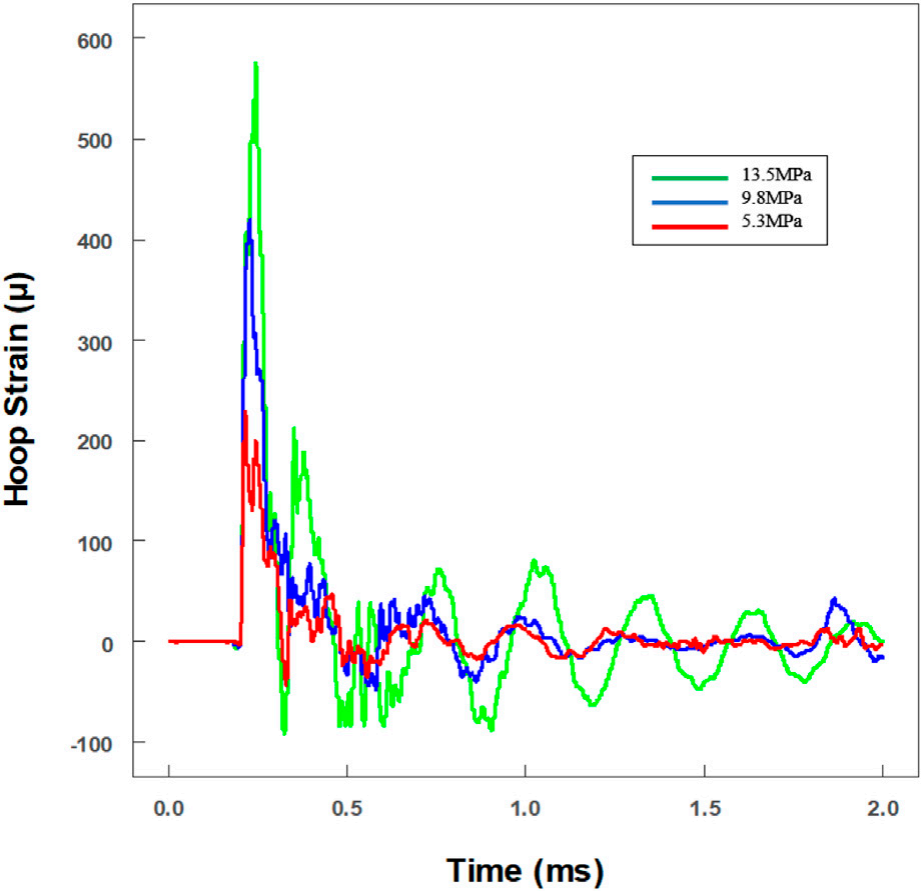
Real-time historical tracing of hoop strain with an extra hollow tube for the *in vitro* cell experiment. The value of strain had been transformed to pressure amplitude. Three different pressures were loaded on cells (green line: 13.5 ± 1.9 MPa; blue line: 9.8 ± 0.9 MPa; and red line: 5.3 ± 0.4 MPa).

To investigate the tolerance ability to dynamic impact loading, the viability of microglia was explored after various levels of impact loading. Real-time hoop strains of three typical pressure levels were recorded. With the linear equation (max hoop strain versus max pressure), the real-time pressures had been calculated (green line: 13.5 ± 1.9 MPa; blue line: 9.8 ± 0.9 MPa; and red line: 5.3 ± 0.4 MPa) (Figure 5). The distance between the strain gauges and the NBR membrane was nearly 74.1 mm, and the reflected wave was predicted at about 1.32 ms after the primary wave signal was first recorded. It is worth noting that the level of the reflected wave was reduced very obviously (<10%) (Figure 5).

**FIGURE 6:**
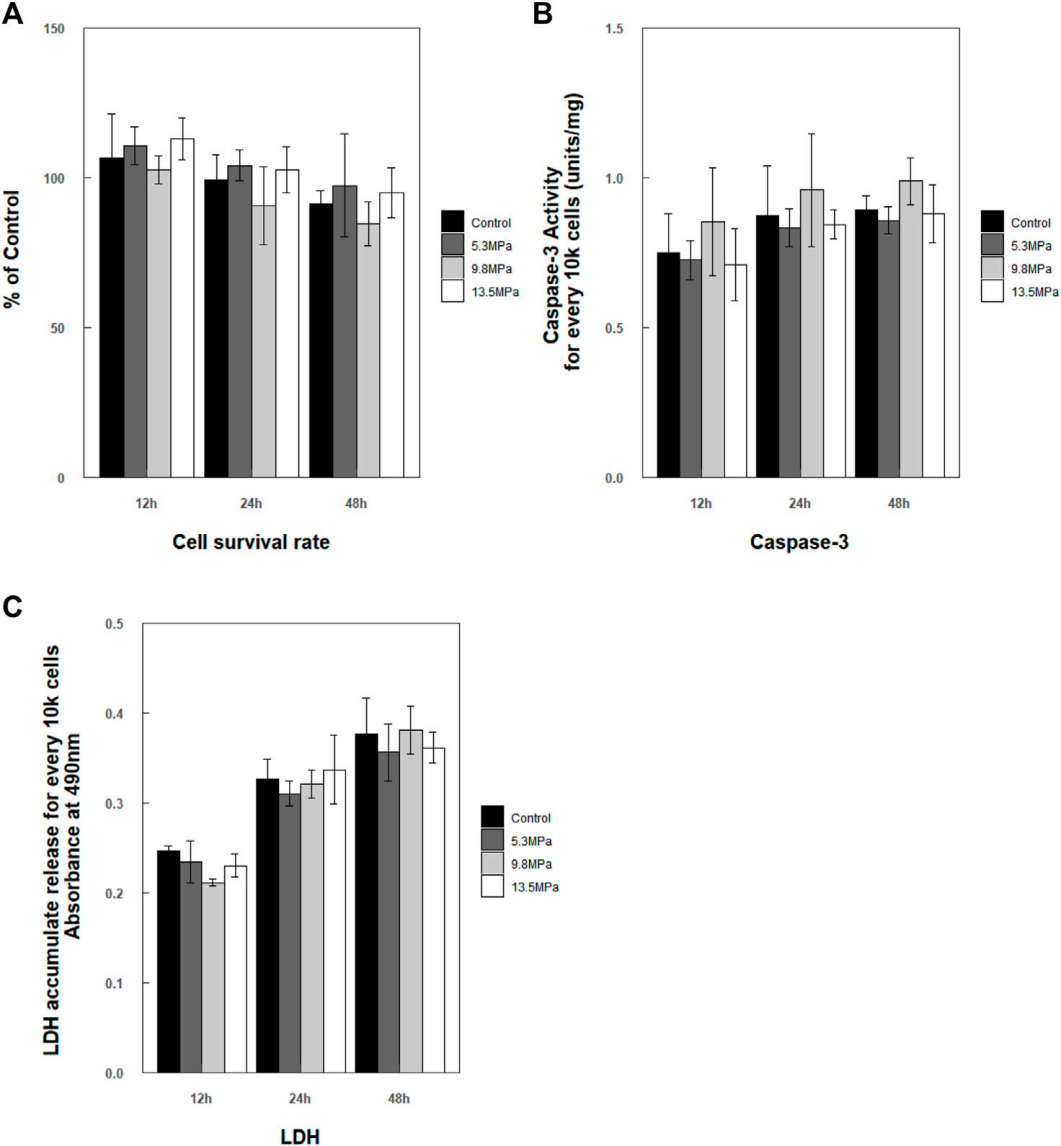
Analysis of cell survival rate within 48 h post exposure. **(A)** Cell survival rate (n=4) ; **(B)** caspase-3 enzyme activation. The values represent the mean units/mg ± S.E.M (n=3) and are normalized for every 10 k cells; **(C)** analysis of accumulated LDH released into the cell medium. The values represent the mean OD value ± S.E.M for every 10 k cells (n=4) . The statistical method is one-way ANOVA with Tukey’s *post hoc* test.

After exposure to impact loading, no apparent morphology changes were observed microscopically (even to 13.5 MPa). Cells maintained their shapes, and no or little debris was noted. Then, CCK-8, caspase-3 enzyme activity, and LDH release of microglia were analyzed at 12, 24, and 48 h, respectively, post exposure (Figure 6). Cell survival rate analysis as well as caspase-3 enzyme activity in the cytoplasm and the accumulated released LDH showed no statistically significant changes compared to control at each time point (Figure 6).

### Stress Response and Cytokine Production

**FIGURE 7:**
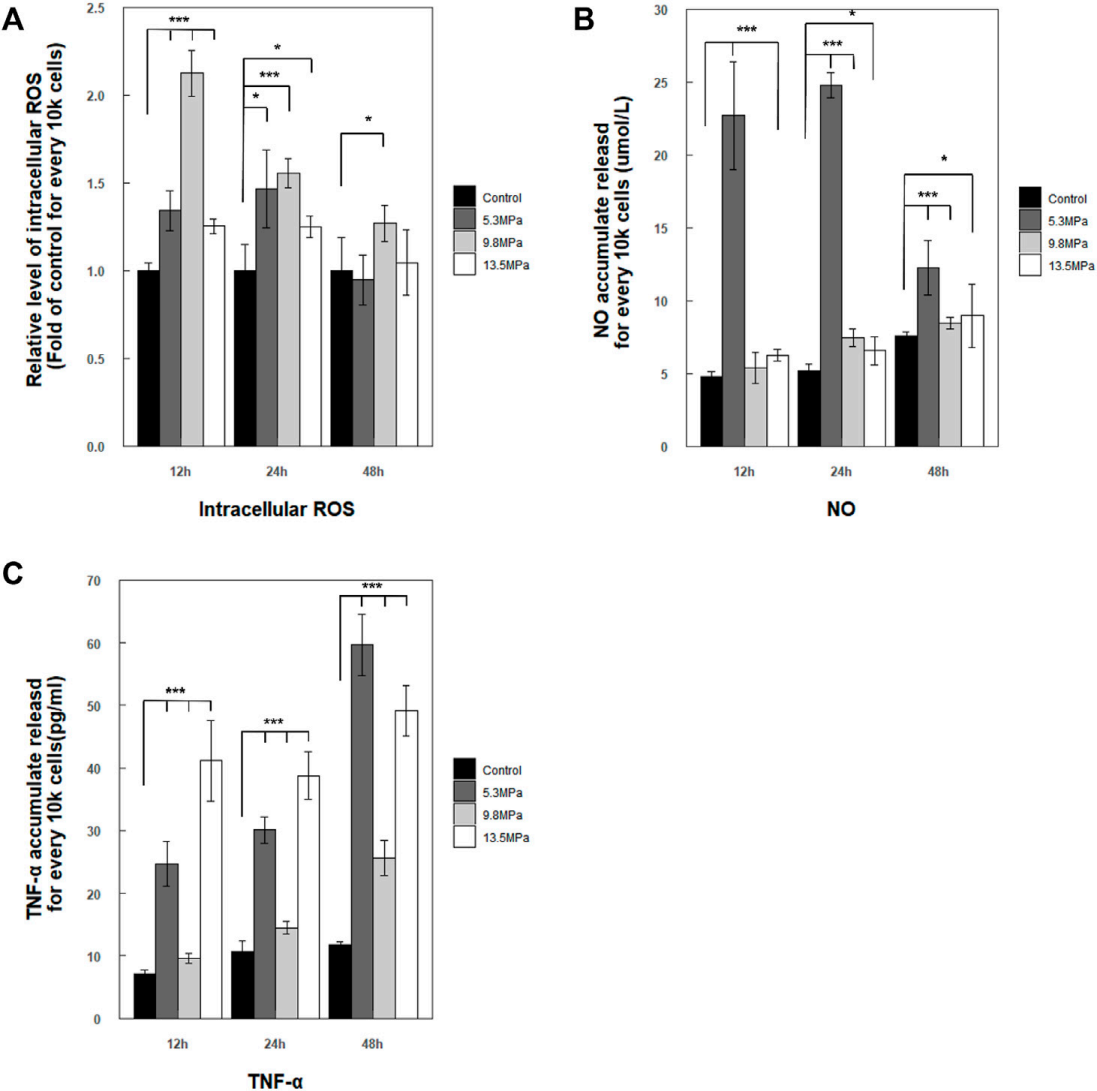
Stress and inflammatory response by primary microglia within 48 h post exposure. **(A)** Accumulated intercellular ROS level regulation was presented as a relative level form (fold of control within each experimental time point) and normalized for every 10 k cells (n=4) ; **(B)** accumulated secretion of NO into the cell medium was presented as a form of mean μmol/L± S.E.M and normalized for every 10 k cells (n=4) ; **(C)** concentration of TNF-α regulation. The concentration was calculated by comparing with the standard curve. The values represent the mean pg/mL± S.E.M and are normalized for every 10 k cells (n=3) . All the results were analyzed by one-way ANOVA followed by Tukey’s test. The comparison only occurred between the experimental groups and control groups within each experimental time point (*p-value less than 0.05 was considered significant; **p-value less than 0.01 was considered significant; and ***p-value less than 0.001 was considered significant).

A fundamental hypothesis of this work was that compression stress could induce immune response in microglia. So, the intercellular ROS and the secretion of NO and TNF-α in the cell medium were measured after applying compression stress (Figure 7). The relative levels of intercellular ROS showed a significant increase and reached peaks at 12 h compared with those of the control group (*p* < 0.001) and then decreased to the nearly same level as the control group within 48 h, except the 9.8 MPa group which still showed a slightly higher level than the control group (p<0.05) (Figure 7A). The released NO in the cell medium of the low-pressure group (5.3 MPa) jumped to a very high level compared to that of the control group (nearly 4 to 5 folds of control) at 12 and 24 h (*p* < 0.001) and then dropped to about 2-fold compared to that of the control group (*p* < 0.001) at 48 h (Figure 7B). As for the moderate-pressure group (9.8 MPa), the released NO exhibited a significant increase at the experimental time points 24 and 48 h (p<0.001) , except the 12 h time point (p>0.05) . In addition, the highest-pressure group (13.5 MPa) also showed a trace increased level of NO compared with the control group at every time point, 12 h (*p* < 0.001), 24 h (*p* < 0.05), and 48 h (*p* < 0.05). The accumulated concentrations of secreted TNF-α after impact loading within 48 h are shown in Figure 7C. After the impact experiments, for all pressure groups, primary microglia were found with a significant increase in the accumulated released results of TNF-α in the cell medium at all time points (p<0.001) .

Taken together, microglia were sensitive to dynamic impact loading with acute stress and inflammatory response; the stress response can be detected early at 12 h and mostly returned to normal within 48 h, while the inflammatory responses of secreted TNF-α were much quicker and stronger after high-pressure impact.

## Discussion

The study on TBI has been carried on for decades, during which many models had been developed to apply impact pressure on animals, tissues, and culture cells, such as shock tube and fluid percussion (Courtney and Courtney, 2010; Kuehn et al., 2011; Pun et al., 2011; Woods et al., 2013; Simard et al., 2014). The main limitation in research is how to generate an accurate and idealized dynamic pressure loading process at physiological scales. This modeling process requires well-considered and clearly described various factors, including stress factors (waveform, amplitude, and duration) (Moeendarbary and Harris, 2014). In addition, when the length scale comes to a single cell or cell monolayer, the reflected waves during stress wave propagating will make the situation more complicated and uncontrollable (Chen and Chen, 2011; Serra-Picamal et al., 2012). Therefore, to minimize such disturbances in the dynamic loading process, based on a simplified FSI model (the theories and model are detailed in the Method part), we have developed a single-pulse dynamic pressure loading system for *in vitro* cells in the laboratory. This system was convenient to generate a compression wave in the fluid with real-time monitoring (Figure 1). Cell suffered the dynamic loading under a normal culture condition, which facilitates the study on behavior response and biomechanical mechanism of the cell after stress *in vitro* (Figures 2, 3). In addition, an extended liquid column was adopted to reduce the reflected waves, as the wave impedance of the NBR membrane is close to the medium (Figure 4) (Chen and Chen, 2011). In addition, duration, amplitude, and waveform of the stress wave were adjustable by changing the length/mass, velocity, or material of the projectile. Theoretically, the maximum amplitude of pressure is the maximum elastic deformation of the tube wall. In this study, a steel projectile was used to generate an exponential-attenuation wave which had been applied to simulate an underwater shock wave (Deshpande et al., 2006).

Microglia play an irreplaceable role in acute response during TBI and very sensitive biological model during pressure loading (Sappington and Calkins, 2008). It was also demonstrated that cells are more sensitive to pressure amplitude than duration (Kane et al., 2012). Then, three different pressure amplitudes (low pressure: 5.3 MPa, moderate pressure: 9.8 MPa, and high pressure: 13.5 MPa) had been applied in this study (Figure 4). The microglia viability after pressure loading within 48 h demonstrated that primary microglia showed very strong tolerance to compression pressure, even at an extremely high-pressure level (13.5 MPa) (Figure 6). It coincides with Sawyers research which performed underwater explosives at 14 MPa (Sawyer et al., 2017a; Sawyer et al., 2017b; Sawyer et al., 2018). Moreover, a similar phenomenon could be found in neuronal/glial cultures (Mukhin et al., 1997). Although most neurons had died, this similar injury causes little glial cell death post 18 h. Under such a microsecond/millimeter scale, the movement of the cell can be negligible, and it can be considered that the cytoskeleton structure of the cell directly bears the compression loading (Ott et al., 1994; Subra, 2007; Sinke et al., 2010). Despite the cytoskeleton structure being quite complex and fluid (Subra, 2007; Hoffman and Crocker, 2009; Tee et al., 2015; Lenne et al., 2021), much research is needed to verify the structural mechanisms of the cytoskeleton on maintaining this stable structure under dynamic loading.

Acute responses of microglia were investigated within 48 h after the pressure loading (Figure 6). The results showed that the pressure loadings have a significant effect on ROS/NO generation and TNF-α cytokine production. The levels of intercellular ROS and the secreted NO reached a peak around 24 h and reduced to normal within 48 h, while the production of TNF-α keeps a high level. These results suggested that cytokine production was likely to have a linearly time-dependent relation. Another noticeable result was the inducible trend of cytokine expression, which did not exhibit a clearly linearly stress-dependent relation; this may be explained by previous reports that a nonlinear relationship may exist between the pressure intensity and the degree of the cellular inflammatory responses (Sawyer et al., 2017a; Sawyer et al., 2017b; Sawyer et al., 2018). In addition, results of acute response also indicate that microglia will rapidly activate the inflammatory response after being loaded by a compression wave and may remain active, thereby prolonging the entire acute inflammatory stress response process. The significantly induced expression of ROS/NO and TNF-α indicated the immune responses of microglia under dynamic loading applied. In addition, a very meaningful aspect was that *in vitro* neurons/glial-cultured trauma models with glutamate antagonists had confirmed by experiments the important role of glutamatergic processes in secondary neuronal injury following CNS trauma, which may have some potential relations with our studies about pure microglial (immediately immunological reactions) (Faden and Simon, 1988; Nilsson et al., 1990; McIntosh, 1993; Morrison et al., 2011). But more questions remain to be addressed to understand how the mechanical signal of the compression wave is received by the microglia and the process of inflammation is quickly initiated.

In summary, this is the first report that a laboratory apparatus provides single-pulse dynamic compression loading to cells *in vitro*. The results presented here provide a rational basis for isolating variables to explore the effects and biomechanical mechanisms of a single mechanical factor on cells. Also, we have highlighted the potential response of microglia during dynamic pressure loading. All these will facilitate the understanding and potential application for immunoregulatory which can be extended to clinical therapy for TBI.

## Data Availability

The raw data supporting the conclusion of this article will be made available by the authors, without undue reservation.

## References

[B1] Atif F. Sayeed I. Ishrat T. Stein D. G. (2009). Progesterone with Vitamin D Affords Better Neuroprotection against Excitotoxicity in Cultured Cortical Neurons Than Progesterone Alone. Mol. Med. 15 (9-10), 328–336. 10.2119/molmed.2009.00016 19603099 PMC2710287

[B2] Bao F. Chen Y. Dekaban G. A. Weaver L. C. (2010). Early Anti-inflammatory Treatment Reduces Lipid Peroxidation and Protein Nitration after Spinal Cord Injury in Rats. J. Neurochem. 88 (6), 1335–1344. 10.1046/j.1471-4159.2003.02240.x 15009633

[B3] Cernak I. Merkle A. C. Koliatsos V. E. Bilik J. M. Luong Q. T. Mahota T. M. (2011). The Pathobiology of Blast Injuries and Blast-Induced Neurotrauma as Identified Using a New Experimental Model of Injury in Mice. Neurobiol. Dis. 41 (2), 538–551. 10.1016/j.nbd.2010.10.025 21074615

[B4] Chen W. Chen W. (2011). Split Hopkinson (Kolsky) Bar. Split Hopkinson (Kolsky) Bar. Boston, MA: Springer.

[B5] Clark D. Perreau V. M. Shultz S. R. Brady R. Lei E. Dixit S. (2019). Inflammation in Traumatic Brain Injury: Roles for Toxic A1 Astrocytes and Microglial–Astrocytic Crosstalk. Neurochem. Res. 10.1007/s11064-019-02721-8 30661228

[B6] Courtney M. W. Courtney A. C. (2010). Note: A Table-Top Blast Driven Shock Tube. Rev. Sci. Instrum. 81 (12), 126103. 10.1063/1.3518970 21198058

[B7] Denny-Brown D. E. Russell W. R. (1941). Experimental Concussion. Proc. R. Soc. Med. 34 (11), 691–692. 10.1177/003591574103401102 19992388 PMC1998140

[B8] Deshpande V. S. Heaver A. Fleck N. A. (2006). An Underwater Shock Simulator. Proc. R. Soc. A 462 (2067), 1021–1041. 10.1098/rspa.2005.1604

[B9] Desprat N. Richert A. Simeon J. Asnacios A. (2005). Creep Function of a Single Living Cell. Biophysical J. 88 (3), 2224–2233. 10.1529/biophysj.104.050278 PMC1305272 15596508

[B10] Doppenberg E. M. R. Choi S. C. Bullock R. (2004). Clinical Trials in Traumatic Brain Injury: Lessons for the Future. J. Neurosurg. Anesthesiol. 16 (1), 87–94. 10.1097/00008506-200401000-00019 14676577

[B11] Faden A. I. Simon R. P. (1988). A Potential Role for Excitotoxins in the Pathophysiology of Spinal Cord Injury. Ann. Neurol. 23 (6), 623–626. 10.1002/ana.410230618 2841902

[B12] Farkas I. Baranyi L. Takahashi M. Fukuda A. Liposits Z. Yamamoto T. (1998). A Neuronal C5a Receptor and an Associated Apoptotic Signal Transduction Pathway. J. Physiology 507 (3), 679–687. 10.1111/j.1469-7793.1998.679bs.x PMC2230831 9508829

[B13] Graham D. I. Mcintosh T. K. Maxwell W. L. Nicoll J. A. R. (2000). Recent Advances in Neurotrauma. J. Neuropathol. Exp. Neurol. 59 (8), 641–651. 10.1093/jnen/59.8.641 10952055

[B14] Hangzhe X. Zhijiang W. Jianru L. Wu H. Peng Y. Fan L. (2017). The Polarization States of Microglia in TBI: A New Paradigm for Pharmacological Intervention. Neural plast. 2017 (3), 5405104. 10.1155/2017/5405104 28255460 PMC5309408

[B15] Hao Y. Cheng S. Tanaka Y. Hosokawa Y. Yalikun Y. Li M. (2020). Mechanical Properties of Single Cells: Measurement Methods and Applications. Biotechnol. Adv. 45, 107648. 10.1016/j.biotechadv.2020.107648 33080313

[B16] Hoffman B. D. Crocker J. C. (2009). Cell Mechanics: Dissecting the Physical Responses of Cells to Force. Annu. Rev. Biomed. Eng. 11, 259–288. 10.1146/annurev.bioeng.10.061807.160511 19400709

[B17] Howe M. S. (1998). Acoustics of Fluid-Structure Interactions. Cambridge, UK: Cambridge University Press.

[B18] Huber B. R. Meabon J. S. Martin T. J. Mourad P. D. Bennett R. Kraemer B. C. (2013). Blast Exposure Causes Early and Persistent Aberrant Phospho- and Cleaved-Tau Expression in a Murine Model of Mild Blast-Induced Traumatic Brain Injury. Jad 37 (2), 309–323. 10.3233/jad-130182 23948882 PMC4126588

[B19] Hutarew G. (1973). Einführung in die Technische Hydraulik. Berlin Heidelberg: Springer.

[B20] Joukowsky N. (1900). Über den hydraulischen Stoss in Wasserleitungsröhren. Impériale des Sciences de St.Pétersbourg. St. Peterbourg: Memoires de I'Aacademie Imperiale des Scriences de, 1–71.

[B21] Junger M. C. it D. F. (1972). Sound, Structures, and Their Interaction. Cambridge, US. MIT Press.

[B22] Kane M. J. Angoa-Pérez M. Francescutti D. M. Sykes C. E. Briggs D. I. Leung L. Y. (2012). Altered Gene Expression in Cultured Microglia in Response to Simulated Blast Overpressure: Possible Role of Pulse Duration. Neurosci. Lett. 522 (1), 47–51. 10.1016/j.neulet.2012.06.012 22698585 PMC3396767

[B23] Korteweg D. J. (2010). Ueber die Fortpflanzungsgeschwindigkeit des Schalles in elastischen Röhren. Ann. Der Phys. 241 (12), 525–542.

[B24] Kuehn R. Simard P. F. Driscoll I. Keledjian K. Ivanova S. Tosun C. (2011). Rodent Model of Direct Cranial Blast Injury. J. Neurotrauma 28 (10), 2155–2169. 10.1089/neu.2010.1532 21639724

[B26] Leishear R. A. (2005). Dynamic Stresses during Structural Impacts and Water Hammer. Columbia, South Carolina: University of South Carolina.

[B25] Leishear R. A. (Editor) (2006). Comparison of Experimental to Theoretical Pipe Strains during Water Hammer. ASME 2006 Pressure Vessels and Piping/ICPVT-11 Conference, Vancouver, BC, Canada, July, 2006.

[B27] Leishear R. A. (2007). Stresses in a Cylinder Subjected to an Internal Shock. J. Press. Vessel Technol. 129 (3), 372–382. 10.1115/1.2748820

[B28] Lenne P.-F. Rupprecht J.-F. Viasnoff V. (2021). Cell Junction Mechanics beyond the Bounds of Adhesion and Tension. Dev. Cell 56 (2), 202–212. 10.1016/j.devcel.2020.12.018 33453154

[B29] Long X. Yao X. Jiang Q. Yang Y. He X. Tian W. (2020). Astrocyte-derived Exosomes Enriched with miR-873a-5p Inhibit Neuroinflammation via Microglia Phenotype Modulation after Traumatic Brain Injury. J. Neuroinflammation 17 (1), 89–15. 10.1186/s12974-020-01761-0 32192523 PMC7082961

[B30] Maas A. I. R. Dearden M. Servadei F. Stocchetti N. Md|| A. U. (2000). Current Recommendations for Neurotrauma. Curr. Opin. Crit. Care 6 (4), 281–292. 10.1097/00075198-200008000-00008 11329513

[B31] Marshall L. F. (2000). Head Injury: Recent Past, Present, and Future. Neurosurgery 47 (3), 546–561. 10.1227/00006123-200009000-00002 10981741

[B32] McIntosh T. K. (1993). Novel Pharmacologic Therapies in the Treatment of Experimental Traumatic Brain Injury: a Review. J. neurotrauma 10 (3), 215–261. 10.1089/neu.1993.10.215 8258838

[B33] Moeendarbary E. Harris A. R. (2014). Cell Mechanics: Principles, Practices, and Prospects. WIREs Mech. Dis. 6 (5), 371–388. 10.1002/wsbm.1275 PMC4309479 25269160

[B34] Morganti-Kossmann M. C. Rancan M. Otto V. I. Stahel P. F. Kossmann T. (2001). Role of Cerebral Inflammation after Traumatic Brain Injury: a Revisited Concept. Shock 16 (3), 165–177. 10.1097/00024382-200116030-00001 11531017

[B35] Morrison B. Elkin B. S. Dollé J.-P. Yarmush M. L. (2011). *In Vitro* Models of Traumatic Brain Injury. Annu. Rev. Biomed. Eng. 13 (1), 91–126. 10.1146/annurev-bioeng-071910-124706 21529164

[B36] Mukhin A. G. Ivanova S. A. Knoblach S. M. Faden A. I. (1997). NewIn VitroModel of Traumatic Neuronal Injury: Evaluation of Secondary Injury and Glutamate Receptor-Mediated Neurotoxicity. J. neurotrauma 14 (9), 651–663. 10.1089/neu.1997.14.651 9337127

[B37] Nguyen H. X. O'Barr T. J. Anderson A. J. (2010). Polymorphonuclear Leukocytes Promote Neurotoxicity through Release of Matrix Metalloproteinases, Reactive Oxygen Species, and TNF-Alpha. J. Neurochem. 102 (3), 900–912. 10.1111/j.1471-4159.2007.04643.x 17561941

[B38] Nilsson P. Hillered L. Pontén U. Ungerstedt U. (1990). Changes in Cortical Extracellular Levels of Energy-Related Metabolites and Amino Acids Following Concussive Brain Injury in Rats. J. Cereb. Blood Flow. Metab. 10 (5), 631–637. 10.1038/jcbfm.1990.115 2384536

[B39] Ott L. Mcclain C. J. Gillespie M. Young B. (1994). Cytokines and Metabolic Dysfunction after Severe Head Injury. J. Neurotrauma 11 (5), 447–472. 10.1089/neu.1994.11.447 7861440

[B40] Patterson L. H. (2020). The MicroHammer: Investigating Cellular Response to Impact with a Microfluidic Mems Device. Santa Barbara: University of California.

[B41] Perotti L. E. Deiterding R. Inaba K. Shepherd J. Ortiz M. (2013). Elastic Response of Water-Filled Fiber Composite Tubes under Shock Wave Loading. Int. J. Solids Struct. 50 (3-4), 473–486. 10.1016/j.ijsolstr.2012.10.015

[B42] Pun P. B. L. Kan E. M. Salim A. Li Z. Ng K. C. Moochhala S. M. (2011). Low Level Primary Blast Injury in Rodent Brain. Front. Neur. 2, 19. 10.3389/fneur.2011.00019 PMC3083909 21541261

[B43] Raghupathi R. (2010). Cell Death Mechanisms Following Traumatic Brain Injury. Brain Pathol. 14 (2), 215–222. 10.1111/j.1750-3639.2004.tb00056.x PMC8096005 15193035

[B44] Readnower R. D. Chavko M. Adeeb S. Conroy M. D. Pauly J. R. Mccarron R. M. (2010). Increase in Blood-Brain Barrier Permeability, Oxidative Stress, and Activated Microglia in a Rat Model of Blast-Induced Traumatic Brain Injury. J. Neurosci. Res. 88 (16), 3530–3539. 10.1002/jnr.22510 20882564 PMC2965798

[B45] Rubovitch V. Ten-Bosch M. Zohar O. Harrison C. R. Tempel-Brami C. Stein E. (2011). A Mouse Model of Blast-Induced Mild Traumatic Brain Injury. Exp. Neurol. 232 (2), 280–289. 10.1016/j.expneurol.2011.09.018 21946269 PMC3202080

[B46] Sappington R. M. Calkins D. J. (2008). Contribution of TRPV1 to Microglia-Derived IL-6 and NFkappaB Translocation with Elevated Hydrostatic Pressure. Invest. Ophthalmol. Vis. Sci. 49 (7), 3004–3017. 10.1167/iovs.07-1355 18362111 PMC4139938

[B47] Sawyer T. W. Josey T. Wang Y. Villanueva M. Ritzel D. V. Nelson P. (2017). Investigations of Primary Blast-Induced Traumatic Brain Injury. Shock Waves 28 (1), 85–99. 10.1007/s00193-017-0756-2

[B48] Sawyer T. W. Lee J. J. Villanueva M. Wang Y. Nelson P. Song Y. (2017). The Effect of Underwater Blast on Aggregating Brain Cell Cultures. J. Neurotrauma 34 (2), 517–528. 10.1089/neu.2016.4430 27163293

[B49] Sawyer T. W. Ritzel D. V. Wang Y. Josey T. Villanueva M. Nelson P. (2018). Primary Blast Causes Delayed Effects without Cell Death in Shell-Encased Brain Cell Aggregates. J. Neurotrauma 35 (1), 174–186. 10.1089/neu.2016.4961 28726571

[B50] Scheiblich H. Dansokho C. Mercan D. Schmidt S. V. Bousset L. Wischhof L. (2021). Microglia Jointly Degrade Fibrillar Alpha-Synuclein Cargo by Distribution through Tunneling Nanotubes. Cell 184 (20), 5089–5106. e21. 10.1016/j.cell.2021.09.007 34555357 PMC8527836

[B51] Schmidt O. I. Heyde C. E. Ertel W. Stahel P. F. (2005). Closed Head Injury—An Inflammatory Disease? Brain Res. Rev. 48 (2), 388–399. 10.1016/j.brainresrev.2004.12.028 15850678

[B52] Scholz M. Cinatl J. Schädel-Höpfner M. Windolf J. (2007). Neutrophils and the Blood-Brain Barrier Dysfunction after Trauma. Med. Res. Rev. 27 (3), 401–416. 10.1002/med.20064 16758487

[B53] Serra-Picamal X. Conte V. Vincent R. Anon E. Tambe D. T. Bazellieres E. (2012). Mechanical Waves during Tissue Expansion. Nat. Phys. 8 (8), 628–634. 10.1038/nphys2355

[B54] Shepard S. R. Ghajar J. B. G. Giannuzzi R. Kupferman S. Hariri R. J. (1991). Fluid Percussion Barotrauma Chamber: a New *In Vitro* Model for Traumatic Brain Injury. J. Surg. Res. 51 (5), 417–424. 10.1016/0022-4804(91)90144-b 1758175

[B55] Shepherd J. E. Inaba K. (2009). Shock Loading and Failure of Fluid-Filled Tubular Structures. Dyn. Fail. Mater. Struct. Chapter 6, 153–190. Boston, MA: Springer. 10.1007/978-1-4419-0446-1_6

[B56] Shohami E. Novikov M. Bass R. Yamin A. Gallily R. (1994). Closed Head Injury Triggers Early Production of TNFα and IL-6 by Brain Tissue. J. Cereb. Blood Flow. Metab. 14 (4), 615–619. 10.1038/jcbfm.1994.76 8014208

[B57] Simard J. M. Pampori A. Keledjian K. Tosun C. Schwartzbauer G. Ivanova S. (2014). Exposure of the Thorax to a Sublethal Blast Wave Causes a Hydrodynamic Pulse that Leads to Perivenular Inflammation in the Brain. J. Neurotrauma 31 (14), 1292–1304. 10.1089/neu.2013.3016 24673157 PMC4108981

[B58] Sinke A. P. Jayakumar A. R. Panickar K. S. Moriyama M. Reddy P. V. Norenberg M. D. (2010). NFkappaB in the Mechanism of Ammonia-Induced Astrocyte Swelling in Culture. J. Neurochem. 106 (6), 2302–2311. 10.1111/j.1471-4159.2008.05549.x PMC2597622 18662246

[B59] Skalak R. (1956). An Extension of the Theory of Water Hammer. Transaction ASME 78 (1), 105–115. 10.1115/1.4013579

[B60] Smith R. T. (1973). Weak Shock Wave Propagation in Liquid Media. London, UK: Imperial College London.

[B61] Subra S. (2007). Biomechanics and Biophysics of Cancer Cells. Acta Mater. 3 (4), 413–438. 10.1016/j.actbio.2007.04.002 PMC2917191 17540628

[B62] Tee Y. H. Shemesh T. Thiagarajan V. Hariadi R. F. Anderson K. L. Page C. (2015). Cellular Chirality Arising from the Self-Organization of the Actin Cytoskeleton. Nat. Cell Biol. 17 (4), 445–457. 10.1038/ncb3137 25799062

[B63] Thompson H. J. Lifshitz J. Marklund N. Grady M. S. Graham D. I. Hovda D. A. (2005). Lateral Fluid Percussion Brain Injury: a 15-year Review and Evaluation. J. Neurotrauma 22 (1), 42–75. 10.1089/neu.2005.22.42 15665602

[B64] Tijsseling A. S. (1996). FLUID-STRUCTURE INTERACTION IN LIQUID-FILLED PIPE SYSTEMS: A REVIEW. J. Fluids Struct. 10, 109–146. 10.1006/jfls.1996.0009

[B65] Trepat X. Grabulosa M. Puig F. Maksym G. N. Navajas D. Farré R. (2004). Viscoelasticity of Human Alveolar Epithelial Cells Subjected to Stretch. Am. J. Physiology-Lung Cell. Mol. Physiology 287 (5), L1025–L1034. 10.1152/ajplung.00077.2004 15246973

[B66] J. C. Veilleux J. E. Shepherd (Editors) (2017). Impulsively-Generated Pressure Transients and Strains in a Cylindrical Fluid-Filled Tube Terminated by a Converging Section. Asme Pressure Vessels & Piping Conference, Waikoloa, Hawaii, July, 2017.

[B67] Walters T. W. Leishear R. A. (Editors) (2018). When the Joukowsky Equation Does Not Predict Maximum Water Hammer Pressures. Pressure Vessels and Piping Conference, Prague, Czech Republic, July, 2018. (American Society of Mechanical Engineers (ASME)).

[B68] Woods A. S. Colsch B. Jackson S. N. Post J. Baldwin K. Roux A. (2013). Gangliosides and Ceramides Change in a Mouse Model of Blast Induced Traumatic Brain Injury. ACS Chem. Neurosci. 4 (4), 594–600. 10.1021/cn300216h 23590251 PMC3629744

[B69] Wylie E. B. Streeter V. L. Suo L. (1993). Fluid Transients in Systems. Englewood Cliffs, NJ: Prentice-Hall.

[B70] Yu S. Zhang H. Hei Y. Yi X. Baskys A. Liu W. (2019). High Mobility Group Box-1 (HMGB1) Antagonist BoxA Suppresses Status Epilepticus-Induced Neuroinflammatory Responses Associated with Toll-like Receptor 2/4 Down-Regulation in Rats. Brain Res. 1717, 44–51. 10.1016/j.brainres.2019.04.007 30986405

